# Computing the effects of temperature and osmotic stress on the seed germination of *Helianthus annuus* L. by using a mathematical model

**DOI:** 10.1038/s41598-024-60015-8

**Published:** 2024-05-01

**Authors:** Maryam Javid, Sami Ullah, Fazal Amin, Wadood Shah, Tabarak Malik, Mona S. Alwahibi, Abdul Waheed, Sezai Ercisli, Baber Ali

**Affiliations:** 1 Department of Botany, University of Peshawar, Peshawar, 25120 Pakistan; 2 Biological Sciences Research Division, Pakistan Forest Institute, Peshawar, 25120 Pakistan; 3 Department of Biomedical Sciences, Institute of Health, Jimma University, 378 Jimma, Ethiopia; 4 Department of Botany and Microbiology, College of Science, King Saud University, 11451 Riyadh, Saudi Arabia; 5 Shenzhen Branch, Guangdong Laboratory for Lingnan Modern Agriculture, Genome Analysis Laboratory of the Ministry of Agriculture, Agricultural Genomics Institute at Shenzhen, Chinese Academy of Agricultural Sciences, Shenzhen, 518124 China; 6 Department of Horticulture, Agricultural Faculty, Ataturk University, 25240 Erzurum, Türkiye; 7 HGF Agro, Ata Teknokent, 25240 Erzurum, Türkiye; 8 Department of Plant Sciences, Quaid-i-Azam University, Islamabad, 45320 Pakistan; 9 Adjucnt Faculty, Division of Research & Development, Lovely Professional University, Phagwara, India

**Keywords:** Germination, Osmotic potential, Hydrothermal time model, Temperature, Plant sciences, Climate sciences, Ecology, Environmental sciences

## Abstract

An extremely important oil crop in the world, *Helianthus annuus* L. is one of the world's most significant members of the Asteraceae family. The rate and extent of seed germination and agronomic features are consistently affecting  by temperature (T) and changes in water potential (ψ). A broad hydrothermal time model with T and ψ components could explain sunflower responses over suboptimal T and ψ. A lab experiment was performed using the HTT model to discover both T and ψ and their interactive effects on sunflower germination and also to figure  out the cardinal Ts values. The sunflower seeds were germinated at temperatures (15 °C, 20 °C, 25 °C and 30 °C); each Ts had five constant ψs of 0, 0.3, 0.6, 0.9, and 1.2 MPa via PEG 6000 as osmotic stress inducer. The results revealed that highest germination index was found in seed grown at 20 °C in distilled water (0 MPa) and the lowest at 30 °C with osmotic stress of (− 1.2 MPa). The highest value of germination rate index was found in seed grown at 20 °C in distilled water (0 MPa) and the lowest at 15 °C with an osmotic stress of (− 1.2 MPa). In conclusion, water potential, temperature, and their interactions have a considerable impact on seed germination rate, and other metrics (GI, SVI-I, GRI, GE, SVI-II, and MGT). Seeds sown  at 20 °C with zero water potential showed high germination metrics such as GE, GP, GRI, and T50%. The maximum value to TTsub noted at 30 °C in − 0.9 MPa osmotic stress and the minimum value was calculated at 15 °C in − 1.2 MPa osmotic stress. The result of TTsupra recorded highest at 15 °C in  controlled group (0 MPa). Moreover, θH was  highest at 30 °C in controlled condition (0 MPa) and minimum value was observed at  20 °C under − 1.2 MPa osmotic stress. The value of θHTT were  maximum at  30 °C in controlled group (0 MPa) and minimum value was  recorded at 15 °C under − 1.2 MPa osmotic potential. The base, optimum and ceiling temperatures for sunflower germination metrics in this experiment were noted  6.8, 20 and 30 °C respectively.

## Introduction

The *Helianthus annuus* L. (Sunflower) is a member of the Asteraceae family. It is one of the world's most important oil crops^1^. *Helianthus annuus* L*.* is the world's fourth-major producer of oil seeds. The English name Sunflower, which was given to these flowers in reference to the idea that they move with the sun during the day, always turning in the direction of its rays, shares the same meaning as the Greek name *Helianthus*, which combines the words helios (the sun) and anthos (flower). Sunflower (*Helianthus annuus* L.) plant stalks that have dried up are burned as fuel^2,3^. Sunflower plant is a wonder oil seed crop that is grown all over the world for its seeds. Researchers have discovered that sunflower seeds, a nutrient-rich meal, may help prevent chronic inflammatory disorders, fungus and bacteria infestations, cardiovascular diseases, skin issues, and possibly cancer. The occurrence of proteins, unsaturated fatty acid, phytosterols and varieties of vitamins are responsible for these advantages of sunflower seeds. Numerous studies have shown that sunflower seeds are effective in treating a range of clinical problems^4^.

The key factor impacting seed dormancy and germination is temperature, which controls dormancy and influences how quickly non-dormant seeds germinate. Three cardinal temperatures; maximum optimal, and minimum, have been acknowledged as defining the temperature array across which a specific specie germination of seed occur^5^. The optimal temperature (To) be the temperature at which germination occurs most quickly, the minimum or base temperature (Tb), and the maximum or ceiling temperature (Tc), at which seeds can germinate. When seeds are dormant, temperature array between Tc and Tb is sensitive to their level of dormancy; it is frequently small in inactive seeds and widens when dormancy is lost. Low Tc values, in particular, are frequently linked to seed dormancy. Seeds whose germination is impeded by warm temperatures demonstrate thermo-inhibition or relative dormancy.^6^. The ideal soil temperature for seed germination is that temperature where a seed population experiences the highest percentage of germination in the short length of time (To)^7^. At temperatures above the optimum range (T > To), physiological changes take place in seeds that slow down germination^8,9^. Thermos inhibition describes this gradual loss in seedling growth in a seed population when soil temperature rise above To. The water potentials and temperatures of the seedbed play a significant role in non-dormant seeds' complex physiological process of germination. In order to accurately explain the time, supra and sub-optimal temperatures have been efficiently incorporated in hydrothermal time (HTT) models for a number of plant species at both sub- and supra-optimal temperatures. The hydrothermal time model is a threshold model that takes germination rates and seed population percentages into account at the same time^10,11^.

Thermal time or heat units can be used to define the timing of germination for temperatures below ideal (between Tb and To). That is, for a particular germination percentage tg, the time multiplied by the T in excess of Tb is a fixed for that portion, θT (g). According to this model, the germination rate (GRg or 1/tg) for a specific seed percentage or fraction is a linear function of Temperature above Tb. The maximum temperature at which seeds can grow is known as the ceiling temperature. In many instances, GRg decreases linearly as T between Tc and To rises. To account for this difference in Tc value, Ellis and Horvitz^12^, Covell*, *et al*.*^13^ and Hardegree^14^ proposed this model. Germination rate rates are inversely connected to how much water potential there is, according to the HTT and temperature (T) are over their base or threshold values. The mathematical basis of the HTT, certain applications in modelling seed dormancy and sprouting behaviour, and prospective future directions are discussed. Temperature is the main environmental cue that governs both germination and dormancy^15^. The TT model can take into account changing temperatures by adding the thermal times determined at each T^16^. In order to represent seed germination patterns, the HTT model was created by combining the aforementioned thermal time and hydro-time models.

This study will investigate mathematical model (based on the temperatures and the osmotic potentials) which would predict the germination responses of seeds of *Helianthus annus*. Therefore, the current study aimed; (1) to estimate the effectiveness of the HTT model to examine seed germination of Sunflower at different Ts and Ψs, (2) To calculate SG at cardinal Ts and various osmotic potentials.

## Materials and methods

### Seeds sowing and treatments

Sunflower (*Helianthus annuus* L. var. S1-278) seeds with a 90% viability rate were gathered from the Nuclear Institute for Food and Agriculture (NIFA), Peshawar, Khyber Pakhtunkhwa, Pakistan. The seeds were kept in plastic bags and utilized in an experiment with a hydrothermal time model. Seeds of the sunflower (*Helianthus annuus* L. var. S1-278) were sterilized in mixture of 95 percent distill water and 5 percent mercuric chloride (HgCl). The seeds were filtered via filter paper after keeping 5 min in a solution. After drying, distilled water was used to clean the seeds. The experiment was performed in Plant Physiology laboratory at  the University of Peshawar's department of Botany from February to June 2022 at varied water potentials and temperatures in a room and incubator setting. For the laboratory experiment, Sunflower (*Helianthus annuus* L. var. S1-278) crop was selected for trial. the seeds were sown in Petri dishes (15 cm size), in different groups labelled as controlled and treated. . To prevent water from evaporating, petri dishes were placed randomly in the incubators while being covered in transparent plastic bags. Seed were considered germinated when they showed evident radicle emergence of more than 2 mm.^17^. Five varying  water potentials (0, 0.3,  0.4,  0.5, and 0.6 MPa) solutions were prepared by adding PEG 6000 from which 10 ml solution was added to each Petri dishes and the seeds were planted at temperatures ranging from 15, 20, 25, and 30 °C.

Using distilled water, an osmotic potential of 0 MPa was produced. According to Michel and Kaufman (1973), the negative osmotic potential levels were generated with PEG (polyethylene glycol 6000; Merck, Germany). On the third day of the experiment, a reading test was given. Each replication contained 10 seeds of each type, for a total of 300 seeds per experiment.

### Hydrothermal time model

After the completion of lab experiment, the radical length and plumule length of all repetitions were measured in centimeters (cm) and calculation were done for different parameters.

#### Thermal-time model

According to this model, the germination rate (GR) is a linear function of temperature above Tb for a specific seed fraction, percentage, or germination period. The minimum temperature where such germination can take place is known as the Tb or base temperature. The optimal temperature (To) is the temperature at which germination happens the quickest. The mathematical models that explain how temperature affects germination patterns^11^.

The model at sub-optimal Temperatures was.

1 $${{\text{TT}}}_{{\text{sub}}} =({\text{T}}-{\text{Tb}})\mathrm{ tg }(\mathrm{at sub}-\mathrm{optimal temperature})$$

The model at supra-optimal Temperatures was.

2 $${{\text{TT}}}_{{\text{supra}}}= ({\text{Tc}}-{\text{T}})\mathrm{ tg} (\mathrm{at supra}-\mathrm{optimal temperature})$$

Rate of germination is inverse to time of emergence of seed, So Eqs. (1) and (2) give Eq. (3);

3 $${\text{GR}} =1/tg = \left(\mathbf{T}-\mathbf{T}\mathbf{b}\right)/\mathbf{t}\mathbf{g}$$

#### Hydro-time model

Hydro time model presented by^10^ and^11^. A seed must reach a point where it can no longer germinate when dried from its fully hydrated form. The base or threshold parameter b will only stop percentage g of the seed number from germinating.

4 $$\theta \mathrm{H }(\mathrm{g }) =\left(\uppsi -\mathrm{\psi b}\right)tg$$

5 $$\mathrm{GR }({\text{g}}) = 1 / tg = \left(\uppsi -\mathrm{\psi b}\right)/\mathrm{\theta H}$$

*θ*H is the hydrotime, GR is the germination rate, $\uppsi$ is the osmotic potential, ψb is the base osmotic potential and tg is the germination time.

#### Hydro-thermal model

Combining *θ*H equations with TT equations yields a *θ*HTT constant for suboptimal temperature^11,18^: To represent germination time courses, this hydrothermal model has performed effectively. (Bradford, 2002).

6 $$\theta \mathrm{HTT }=\left(\mathrm{\psi b}-\uppsi \right)\left(\mathrm{T }-{\text{Tb}}\right){\text{tg}}$$

*θ*HTT is the Hydrothermal time model constant, T is the temperature, Tb is the base temperature $\uppsi$ is the osmotic potential, ψb is the base osmotic potential and tg- germination time.

### Germination parameters

#### Mean germination time (MGT)

The population of seeds' MGT represents how quickly they germinated. The mean germination time decreases as the population grows more germinated^19^.

7 $${\text{MGT}} = \sum {{\text{fx}}} /\sum {\text{f}}$$

f is the number of germinated seeds and x is number of days taken to germination.

#### Germination energy (Ge)

We calculated germination energy using a standard method of^20^

8 $${\text{GE}} = {\text{X1}}/{\text{Y1 }} + \left( {{\text{X2}} - {\text{X1}}} \right)/{\text{Y2 }} \ldots \, + \, \left( {{\text{Xn}} - {\text{Xn}} - {1}} \right)/{\text{Yn}}$$

#### Mean germination rate (MGR)

The average rate of germination is equal to the mean germination time reciprocal^21^.

9 $${\text{Mean germination rate }} = { 1}/{\text{Mean germination time}}$$

#### Germination rate index (GRI)

Greater and maximum GR are indicated by higher GRI values; GRI represents the age-percentage of seed germination regularity during the germination stage^22^.

10 $${\text{Germination Rate Index }} = {\text{G1}}/{2 } + {\text{ G2}}/{2 } \ldots \, + {\text{ Gn}}/{2}$$

#### Germination Index (GI)

A systematic calculation method was used to determine the germination index^23^.

11 $${\text{GI }} = \, \left( {{1}0 \, \_{\text{ n1}}} \right) \, + \, \left( {{9 }\_{\text{ n2}}} \right) \, + \, \cdots \, + \, \left( {{1 }\_{\text{ n1}}0} \right)$$

#### Seed vigor index-I (SVI-I)

SVI regarding the seedling’s length by^19^.

12 $${\text{Seed vigor index }} = {\text{ Seedling length }} \times {\text{ germination }}\%$$

#### Seed vigor index-II (SVI-II)

Seed vigor index regarding the seedling dry weight by^19^.

13 $${\text{Seed vigor index }} = {\text{ seedling dry weight }} \times {\text{ germination }}\%$$

### Antioxidants enzymes

#### Guaiacol peroxidase (GPX) activity

We have determined the activity of GPX according to the protocol of Castillo et al.^24^. Fresh plant tissues were mixed in an ice bath at 4 °C with 10 ml of phosphate buffer (pH 7.5) containing 0.5 mM EDTA. 0.1 ml of the centrifuged supernatant was mix with 16 mM guaiacol, 50 mM phosphate buffer (pH 6.1) and 2 mM H_2_O_2_. At 470.0 nm, enzyme activity was measured.

#### Ascorbate peroxidase (APX) activity

The ascorbate peroxidase activity in fresh plant material was assessed using the standard protocol of Nakano and Asada^25^. In a 10 ml phosphate buffer solution (pH 7.5), 500 mg fresh plant material was homogenized. Ascorbic acid 0.5 mM, potassium phosphate buffer (pH 7.0) and EDTA 0.1 mM were added to 0.1 ml of supernatant. By adding 0.1 ml of H_2_O_2_ to the solution, the reaction was initiated. The decrease in OD was measured by spectrophotometer at 290.0 nm.

#### Catalase (CAT) activity

Fresh plant material was tested for catalase (CAT) activity using the standard protocol of Aebi^26^. A 0.5 g plant tissues were homogenized with 10 ml phosphate buffer (pH 7.5) contain 0.5 mM EDTA. Homogenates were filtered, centrifuged for 25 min at 15,000 rpm, and supernatants were collected for catalase activity. 0.1 ml of supernatant was treated with 2 ml of phosphate buffer (1 M) and 0.5 ml of H_2_O_2_ (75 mM). The optical density was measured at 240.0 nm.

#### Superoxide dismutase (SOD) activity

Fresh plant material was analyzed according to the standard protocol of Beauchamp and Fridovich^27^. This sample was prepare by chopping 500 mg of plant material into 5 ml of phosphate buffer. A combination of EDTA (3 mM), nitrotetrazolium blue chloride (NBT; 2.25 mM), 5 ml methionine (390 mM), and 1.5 M Na_2_CO_3_ was added to the supernatants. After 20 min of incubation under ordinary light, 1 ml of riboflavin was added to the reaction mixture. An optical density (OD) measurement was performed with a spectrophotometer at 560.0 nm for the reaction mixture.

#### Peroxidase (POD) activity

Peroxidase (POD) activity in fresh plant tissues was measured according to the protocol of Vetter et al.^28^. Using mortar and pestle and an ice bath, 500 mg of plant material was mixed with 2 ml MES. After centrifuging the mixture at 10,000 rpm for 20 min, the supernatant was collected. One drop of H_2_O_2_ (30%), 1.5 ml of MES buffer (pH 5.5), and 0.1 ml of phenylenediamine were added to 0.1 ml of the supernatant mixture. In this experiment, the OD was measured at 485.0 nm.

### Statistical analysis

For statistical analysis IBM SPSS Statistics 26 and Sigma Plot Version 11.0 software was used. Linear regression was used to examine effects of hydro time, TT and their interactions on germination characteristics and seed germination rate. The values of the parameters ($\uppsi$ (50), $\uppsi$b; R^2^, Sig, F, SE, and T-test) were calculated using linear probate regression analysis in SPSS. The Tukey HSD test was used to show the significant difference between groups Using Excel software, several graphs of the fraction of germination vs the accelerated ageing duration and the germination parameters against $\uppsi$ and T were plotted.

### Ethical approval and consent to participate

Sunflower (*Helianthus annuus* L. var. S1-278) seeds were collected  from the Nuclear Institute for Food and Agriculture (NIFA), Peshawar, Khyber Pakhtunkhwa, Pakistan. The trial was performed in accordance with relevant guidelines and regulations.

## Results

**Figure 1:**
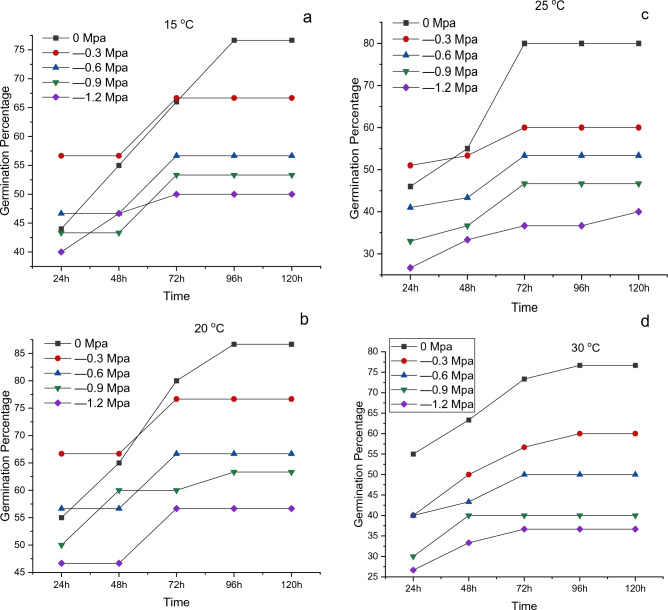
Cumulative germination for *Helianthus annuus* L var. S1-278 at (**a**) 15 °C, (**b**) 20 °C, (**c**) 25 °C and (**d**) 30 °C having different water potentials (0, − 0.3, − 0.6, − 0.9 and − 1.2 MPa). Symbols indicate water potential and lines indicate cumulative germination predicted by the hydrothermal time model.

**Figure 2:**
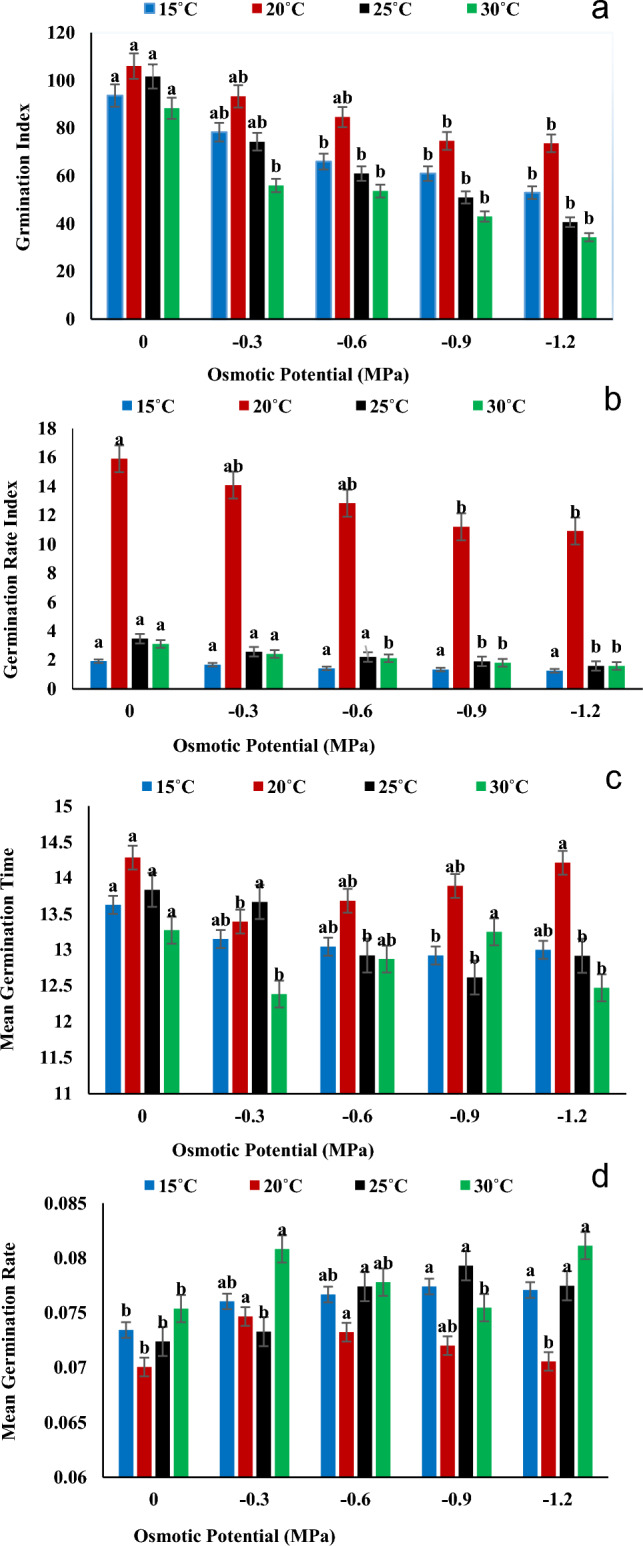
Interactive effect of temperature and water potential on (**a**) Germination Index, (**b**) Germination rate Index, (**c**) Mean Germination Time and (**d**) Mean Germination Rate of *Helianthus annuus* L var. S1-278 using hydrothermal time model. Bars sharing different letter (s) for each parameter are significantly different from each other according to the HSD test (p ≤ 0.05). All the data represented are the average of three replications (n = 3). Error bars represent the standard errors (SE) of three replicates.

Temperature, osmotic potential, and their interactions were found to have a substantial impact on germination rate and other characteristics. According to Fig. 1a–d, The maximum percentage of germination was found in seeds grown in distilled water (0 MPa for each temperature 15 °C, 20 °C, 25 °C and 30 °C and the lowest at (− 1.2 MPa) at 15 °C, 20 °C, 25 °C and 30 °C. According to Fig. 2a, for *Helianthus annuus* L. var. S1-278, the highest germination index was found at 20 °C in distilled water (0 MPa), and the lowest at 30 °C with an osmotic potential of (− 1.2 MPa). The highest value of germination rate index was found at 20 °C in distilled water (0 MPa), and the lowest at 15 °C with an osmotic stress of (− 1.2 MPa) (Fig. 2b). According to Fig. 2c the maximum value of mean germination time was found at 20 °C in distilled water (0 MPa), and the lowest at 15 °C with an osmotic potential of (− 1.2 MPa) (Fig. 2c) and the highest results of mean germination rate were recorded lowest at 30 °C with an osmotic stress of (− 1.2 MPa) while the lowest recorded for 20 °C with distal water (0 MPa) (Fig. 2d).

**Figure 3:**
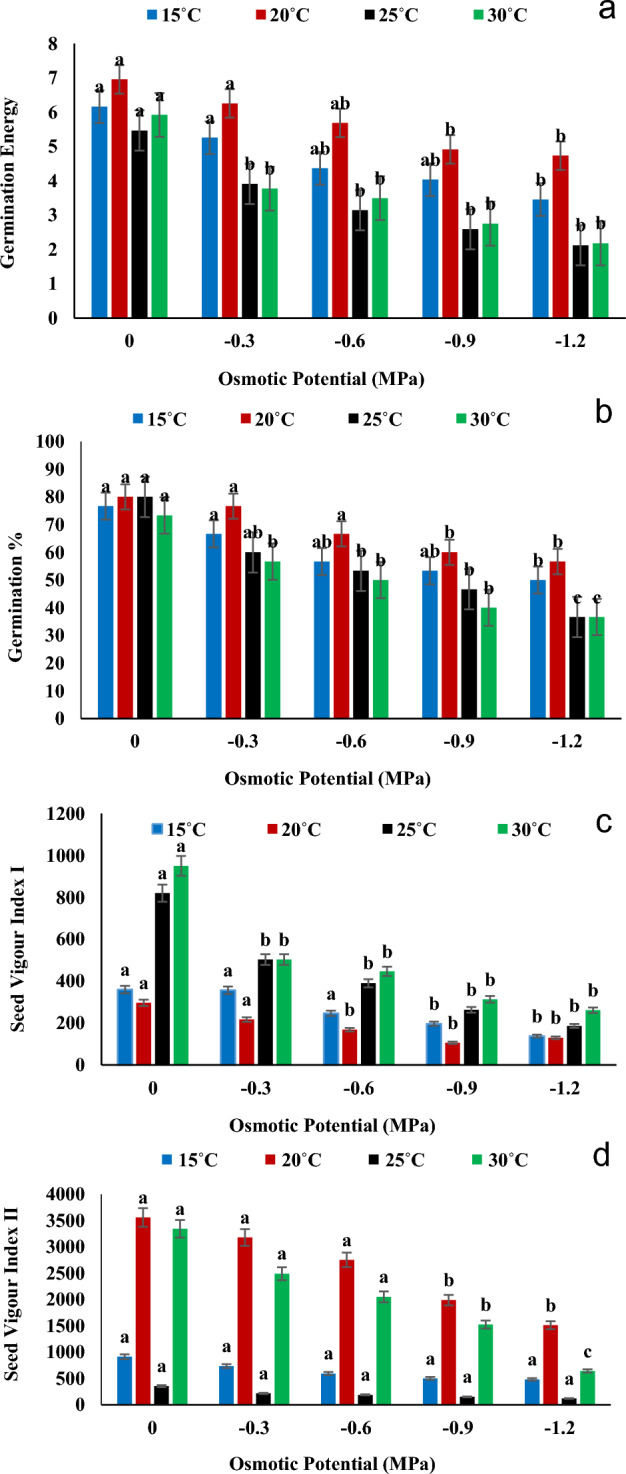
Interactive effect of temperature and water potential on (**a**) Germination Index, (**b**) Timson Germination Index, (**c**) Germination Rate Index and (**d**) Mean Germination Time of *Helianthus annuus* L var. S1-278 using hydrothermal time model. Bars sharing different letter (s) for each parameter are significantly different from each other according to the HSD test (p ≤ 0.05). All the data represented are the average of three replications (n = 3). Error bars represent the standard errors (SE) of three replicates.

**Figure 4:**
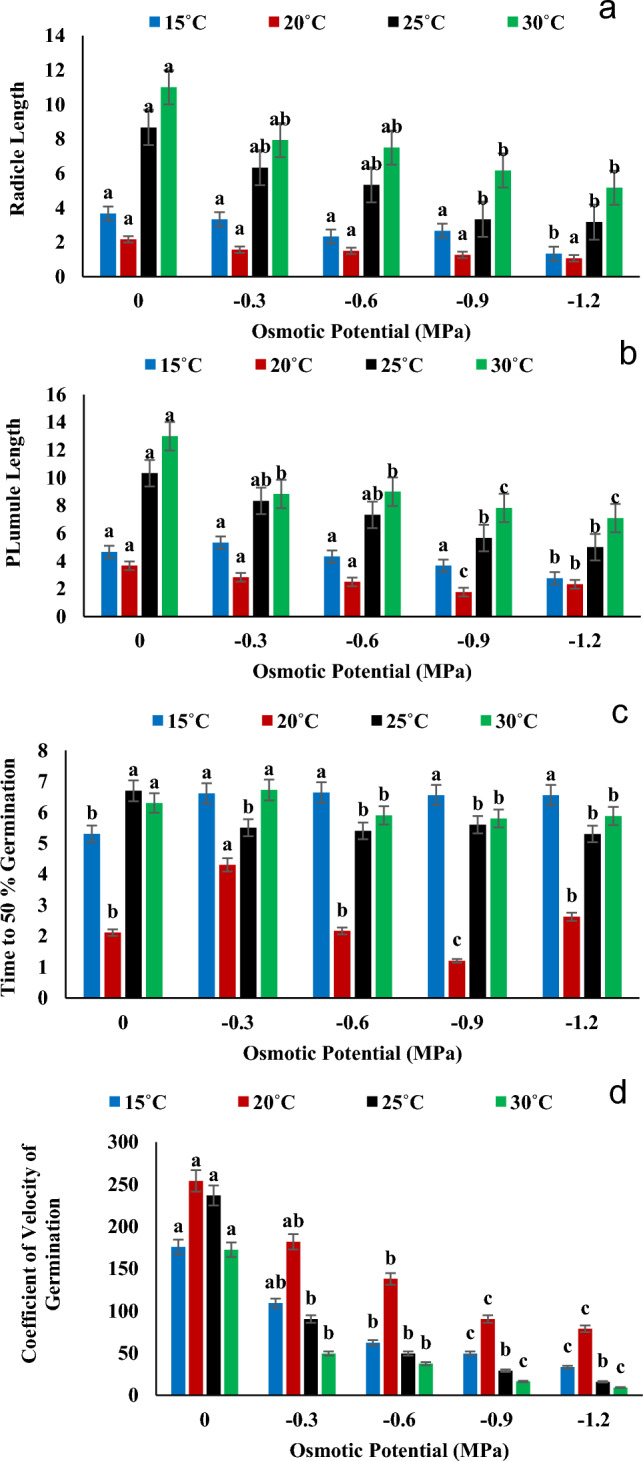
Interactive effect of temperature and water potential on (**a**) Radicle Length, (**b**) Plumule Length, (**c**) Time to 50% Germination and (**d**) Coefficient of Velocity of Germination of *Helianthus annuus* L var. S1-278 using hydrothermal time model. Bars sharing different letter (s) for each parameter are significantly different from each other according to the HSD test (p ≤ 0.05). All the data represented are the average of three replications (n = 3). Error bars represent the standard errors (SE) of three replicates.

The result showed that germination is negatively affected by high osmotic stress (− 1.2 MPa). According to the results recorded in Fig. 3a, it was observed  that germination energy has highest value for distilled water (0 MPa) while the lowest value recorded for − 1.2 MPa osmotic stress at 25 °C. Therefore, osmotic stress reduced the value of germination energy as compared to controlled group. Highest value of overall germination percentage of experiment was recorded for controlled group (0 MPa) at  20 °C while lowest value recorded at 30 °C in − 1.2 MPa osmotic potential (Fig. 3b). According to Fig. 3c, the highest value of seed vigor index-I recognized for distilled water (0 MPa) in 30 °C. While the lowest value recognized at  20 °C for − 0.9 MPa osmotic stress. The seed vigor index-II gave  maximum value in  controlled group at  20 °C and the minimum value was recorded for − 1.2 MPa 25 °C (Fig. 3d). The highest values of radicle length and plumule length were recorded at 0 MPa in 30 °C, while the lowest values were recorded at − 1.2 MPa at 20 °C. The highest time to 50 percent germination was reported in 0 MPa at  25 °C and the maximum value of coefficient of velocity of germination recorded under 0 MPa at  20 °C (Fig. 4a–d).

**Figure 5:**
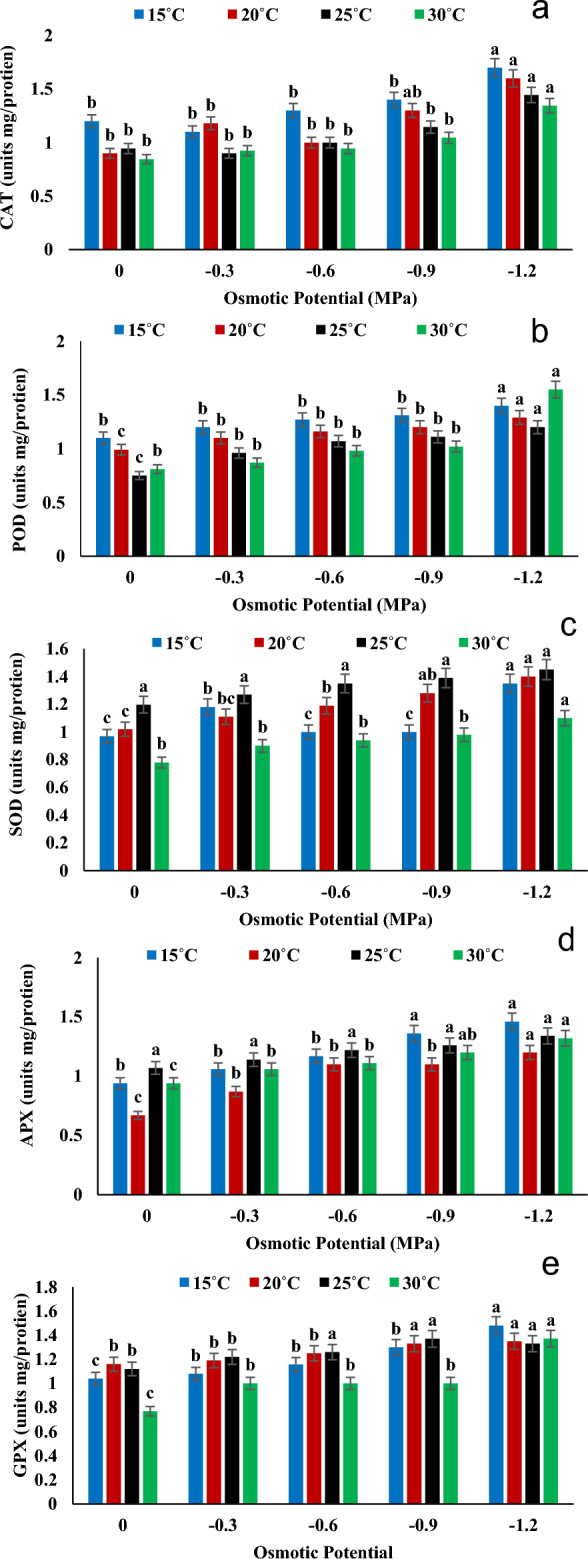
Interactive effect of temperatures and water potentials on (**a**) Catalase activity, (**b**) Peroxidase activity (**c**) superoxide dismutase (**d**) Ascorbate peroxidase and (**e**) Guaiacol peroxidase activity of *Helianthus annuus* L var. S1-278 using hydrothermal time model. Bars sharing different letter (s) for each parameter are significantly different from each other according to the HSD test (p ≤ 0.05). All the data represented are the average of three replications (n = 3). Error bars represent the standard errors (SE) of three replicates.

According to the results of antioxidant enzymatic activities (Fig. 5) temperature and osmotic potential fluctuation highly affected the enzymes' activities and concentration. The highest activity of CAT, APX and GPX was measured at 15 °C in − 1.2 MPa and the lowest value of CAT and GPX were recorded at 30 °C in controlled group (0 MPa). While the APX minimum value was noted at 20 °C under  0 MPa osmotic stress. In addition, highest value of POD was recorded at 30 °C under − 1.2 and the lowest value was observed at  25 °C in controlled condition. Moreover,  highest value of SOD was noted at 25 °C under − 1.2 MPa while the lowest value was measured at 30 °C under  controlled condition. All the enzymatic activities were greatly  affected by both the varying temperature and osmotic potential. The lowest response of all enzymes was recorded at 0 MPa and the highest at  − 1.2 MPa (Fig. 5).

**Figure 6:**
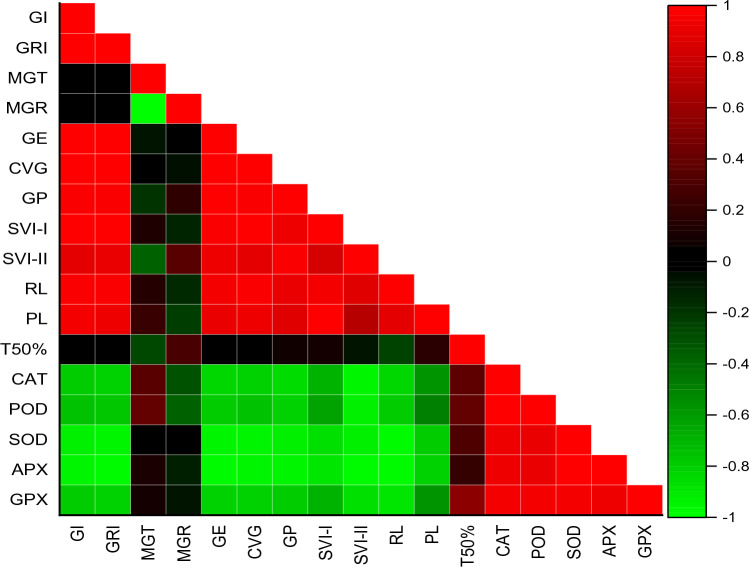
Correlation between various germination attributes of *Helianthus annuus* L var. S1-278 using hydrothermal time model.

**Figure 7:**
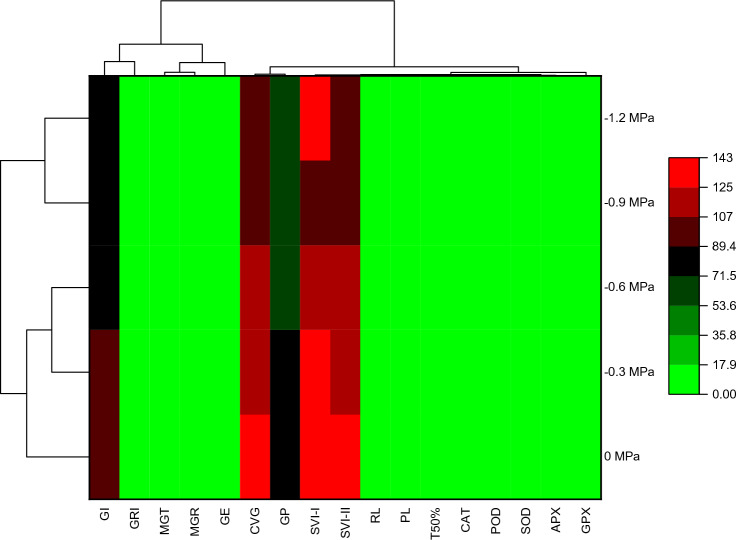
Heatmap histogram correlation between various germination attributes of *Helianthus annuus* L var. S1-278 using hydrothermal time model.

**Figure 8:**
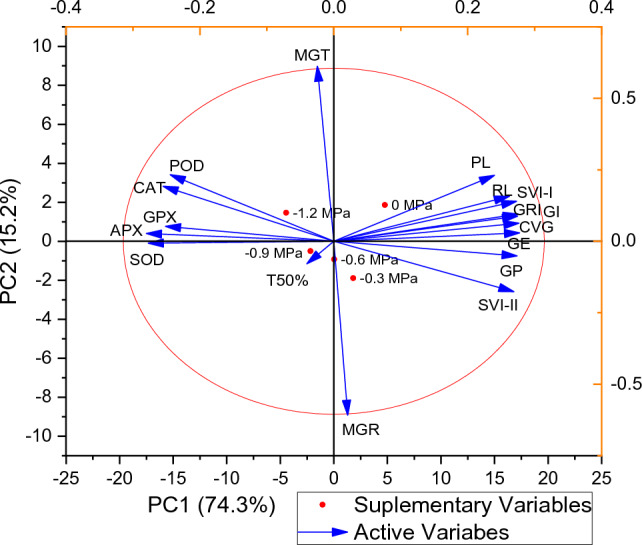
Loading Plot of Principal component analysis (PCA) on various germination attributes of *Helianthus annuus* L var. S1-278 using hydrothermal time model.

The correlation between seventeen measured parameters across five treatments were shown in Fig. 6. According to correlation matrix the SVI-I, GRI, GE, CVG, GP, GI, SVI-II, RL, PL and CVG were positively correlated to each other. MGR was negatively correlated  with GP, SVI-II and T50%. GE has shown negative correlation with PL, RL and SVI-I. All enzymes have  negative correlation with germination parameter while positively correlated with each other. According to Fig. 7 two different clustered were formed between treatments. First cluster consisted of − 0.9 and − 1.2 MPa treatments while the second cluster consisted of controlled group, − 0.3 and − 0.6 MPa osmotic potentials respectively. GI, SVI-II, SVI-I and CVG showed high association with controlled group, − 0.3 and − 0.6 MPa osmotic potentials. PCA was  carried out for the germination of datasets. According to the results, all treatments were variously  distributed in the datasets. The distribution of treatment showed that the osmotic potential significantly affected the germination attributes. The PCA further results described  that the first two component accounted for 74.3% of the total variation. In addition, the first two component showed highest percentage of variance (Fig. 8).

Under sub and supra optimal conditions, the thermal time model effectively predicted germination kinetics for the osmotic potential ranges (0 to − 1.2 MPa) and varying temperatures. Additionally, the TT model had a growing R^2^ value and was holding onto the germinating rate data under filtered water. Furthermore, the ceiling, optimum, and base temperatures were determined using GR (g) responses. The results recorded in Table 1 shows that the highest value to TTsub was noted at 30 °C in − 0.9 MPa osmotic stress and the minimum value was calculated at 15 °C under − 1.2 MPa. The results of TTsupra was recorded highest at 15 °C for controlled group (0 MPa). While the results of θH were highest at  30 °C in controlled group (0 MPa) and minimum value was recorded at  20 °C under − 1.2 MPa. The value of θHTT was  maximum at 30 °C under  controlled conditions (0 MPa) and minimum at  15 °C under − 1.2 MPa osmotic potential. For GR the maximum value was noted under  30 °C for − 1.2 MPa osmotic stress and the minimum value recorded in 30 °C for control (0 MPa). Meanwhile the highest value recorded in 15 °C for − 0.3 MPa osmotic stress and lowest at 25 °C under − 1.2 MPa osmotic stress.

**Table 1:** The estimated parameters of the hydro and thermal time models to describe *Helianthus annuus* L. seed germination under fluctuating different temperatures (Ts) and water potentials (ψs).

Temperature (°C)	Osmotic potential	TTsub/θT1 (Ch^−1^)	TTsupra/θT2 (Ch^−1^)	θH (MPah^−1^)	θHTT (MPa˚Ch^−1^)	TT GR	HT GR
15	0 MPa	187.2	1123.2	56.16	280.8	0.027562	0.027562
	− 0.3 MPa	129.6	777.6	38.58	155.52	0.039195	0.031608
	− 0.6 MPa	97.6	585.6	28.68	87.84	0.052403	0.03213
	− 0.9 MPa	79.2	475.2	22.86	47.52	0.066032	0.027562
	− 1.2 MPa	89.6	537.6	25.68	26.88	0.058544	0.012311
20	0 MPa	272	680	40.8	408	0.03736	0.03736
	− 0.3 MPa	219.2	548	32.88	263.04	0.046151	0.036921
	− 0.6 MPa	192	480	28.8	172.8	0.054653	0.032792
	− 0.9 MPa	179.2	448	26.88	107.52	0.060524	0.02421
	− 1.2 MPa	137.6	344	20.64	41.28	0.073142	0.014628
25	0 MPa	631.2	841.6	63.12	946.8	0.023844	0.023844
	− 0.3 MPa	652.8	870.4	65.28	783.36	0.023188	0.01855
	− 0.6 MPa	520.8	694.4	52.08	468.72	0.02959	0.017754
	− 0.9 MPa	391.2	521.6	39.12	234.72	0.038659	0.015464
	− 1.2 MPa	381.6	508.8	38.16	114.48	0.039308	0.007862
30	0 MPa	995.2	746.4	74.64	1492.8	0.020141	0.020141
	− 0.3 MPa	988.8	741.6	74.16	1186.56	0.020331	0.016265
	− 0.6 MPa	726.4	544.8	54.48	653.76	0.027898	0.016739
	− 0.9 MPa	460.8	345.6	34.56	276.48	0.043489	0.017395
	− 1.2 MPa	422.4	316.8	31.68	126.72	0.047947	0.009589

According to Table 2 the T and ѱ have highly affected germination of sunflower seeds. The thermal time model shown the germination kinetics for increasing temperature at sub and supra-optimal condition. . The minimum value of base water potential at 50 percent germination was  (− 1.01) at  20 °C. The high temperatures (25 °C and 30 °C) have significant value for osmotic potentials (between − 0.6 to − 0.9 MPa). The F value have fluctuations and have no fixed pattern. The base, optimum and ceiling temperatures for sunflower in this experiment were  6.8, 20 and 30 °C respectively (Table 3).

**Table 2:** Estimation of hydrotime model parameters for *Helianthus annuus* L using non-linear regression.

Temperature	ѱb (50) (MPa)	σψb (MPa)	R	R^2^	SD	F	Sig
15c	− 0.89	0.16	0.47	0.66	1.07	3.47	1.14
20c	− 1.01	0.18	0.71	0.85	0.79	5.78	0.63
25c	− 0.82	0.22	0.93	0.96	0.39	26.49	0.16
30c	− 0.78	0.24	0.88	0.93	0.51	17.94	0.26

**Table 3:** Estimated germination and cardinal temperature values for *Helianthus annuus* L. using the hydrothermal time model.

Variables	*Helianthus annuus* L
Hydrothermal time model parameters	
* Ѱ*b (50) (MPa)	− 0.87
σ*ψ*b (MPa)	0.20
θH (MPa˚Ch^−1^)	56.43
kT (MPa˚Ch^−1^)	0.104
Cardinal temperatures	
* T*b (°C)	6.8
* T*o (°C	20
* T*c (°C)	30
* R*^2^	0.851

*kT* Thermal energy of the seed, *Tb* base temperature of the seed, *To* optimum temperature of the seed, *Tc* ceiling temperature of the seed.

## Discussion

Seed lot response to T and ѱ during germination can be quantitatively characterized by the HTT model. Temperature and water potential influences on seed germination can be described and quantified using the HTT model^11^*.* However, numerous investigations reported the tg at sub-optimal TT using the thermal time model. Moreover, these models started to lose effectiveness at supra-optimal TT. Furthermore, the decrease in germination rate when Ts surpassed the ideal temperature was not predicted by thermal time models^11^. In order to overcome these constraints and address associated issues,^11^ and^10^ created the HT and hydrothermal time models. Bradford further claimed that HTT models were reliable tools for comprehending the interactions between Ts and Ys (environmental parameters) in a seed lot as they relate to seed germination^29^

Analyzing germination characteristics under different environmental condition is necessary to pinpoint the best location for a species to flourish and germinate. To measure how these stresses affect seed germination in this way, mathematical model like the HT, TT and HTT model are helpful^30^. Environmental temperature is one of the major environmental factor that influences seed germination in a variety of plant species^31–33^. Another abiotic stress that prevents young seedlings and seeds from sprouting is water stress^34^. Hydrothermal time (HTT), thermal (TT), and Hydrotime (HT) models were created by several research as efficient ways to characterize and forecast seed population germination reactions under various environmental variables. These models are frequently employed as tools in both agronomy and elementary research since they are simple to use and provide a clear biological understanding of the parameters^35^.

According to the findings in Figs. 1, 2 and 3, a substantial decline in growth was observed when water potential was reduced in the germination energy, mean germination time, and germination % at the optimal temperature of 30 °C. The result is similar according to^30^ who hypothesized that variations in water potential would affect FGP more so than changes in temperature. The Sunflower showed least growth at 35 °C which is similar according to the result of^36^ who showed that the growth of agronomic parameters was significantly decrease at 35 °C and almost no growth at 40 °C. The result is also similar to^35^ who displayed the lowest hydrotime value (− 0.6 MPa h) at 35 °C. Our results were also in agreement with the findings of^37^ who reported that the germination of Cucumber and Melon seed were  significantly inhibited at temperatures of 42 °C (8–10%) and 45 °C (9–10%), respectively. Additionally, Watermelon, Winter Squash, Summer Squash, and Pumpkin seeds did not germinate at 42 °C. The T and ѱ had significantly affected the growth of Sunflower. Osmotic potential, temperature, and their interactions had a significant impact on all cultivars' germination rates and germination percentages (P ≤ 0.01)^38^. T and ѱ have a substantial impact on agronomic parameters, water deficit causes a reduction in the pace and percentage of germination; if not a full inhibition^39^.

It was found that drought stress significantly reduced antioxidant enzymes levels, including superoxide dismutase (SOD), peroxidase (POD) and catalase (CAT). Plants subjected to water deficit stress showed a decrease in APX and GPX levels. According to^40^ and^41^, who studied a remarkable increase in antioxidant enzymes levels under abiotic stress, their results were consistent with the findings of our study. Furthermore, it has also been found that the level of antioxidant enzymes in plants was significantly correlated with the level of stressors in the environment, thus scavenging excess oxidative stress and reducing the oxidative damage caused by reactive oxygen species.

The decrease in GP may be because of the heat breakdown of essential amino acids necessary for seed germination with decrease in the concentration of GP a decline in water potential and an equal decrease in water resources may occur^42^. The minimal temperature (Tb) for sunflowers in our study was 15 °C. Tb is a critical cardinal temperature threshold under which the rate of germination drops and plant's physiological activities could be more challenging to maintain, therefore it must be taken into consideration while developing a crop simulation model and selecting the appropriate growth season^11^. Under laboratory conditions, seeds are typically incubated at a constant temperature, but in the field, temperature variations may become a problem at the seedlings' emergence stage. Compared to seeds exposed to steady temperature, those subjected to variable temperatures collected fewer thermal hours^43^. Both the ideal (To) and ceiling (Tc) temperatures for the current study were measured at 30 °C. Cardinal temperatures are roughly three in number: the optimal temperature (To), where high germination happens right away; the ceiling (Tc) and base (Tb) temperatures, where germination rates are at their lowest levels^44^. The ƟH values were used by scientists and breeders to rank cultivars according to how susceptible they were at 30 °C, the base water potential with the lowest 50% germination value (0.18) was measured and it was further shown by Tb constancy that this modification was maintained at all possible temperatures and osmotic potential values. A seed lot's uniform fluctuations in seed germination are represented by the ѱb (g) indicator^45^. Studies on the carrot and onion^46^, potato^17^, tomato^47^ and watermelon^30^ have shown that at optimal temperature, base osmotic potential levels are minimum, and they linearly rise at supra-optimal temperatures.

## Conclusion

In conclusion, water potential, temperature, and their interactions have a considerable impact on germination percentage, germination rate, and other metrics (SVI-I, GRI, GI, SVI-II, MGT and GE). The sunflower germination response might very well be reported by the hydrothermal time models at all osmotic potentials and temperatures. The models correctly predicted (Sunflower) *Helianthus annuus* L. var. S1-278 germination time in response to water potential and temperature. Seeds cultivated at 20 °C with no osmotic potential were shown to have high germination metrics such GE, GP, GRI, and T50%. Therefore, the temperatures, hydrothermal time model (HTT) cardinal and germination findings may be used to illustrate how seed germination would react to changing temperature, individually or together, in upcoming climatic changes. To forecast future germination time courses, the model's parameters should explore how various abiotic stressors affect the physiological behavior of seed populations.

### Supplementary Information

Supplementary Information.

## Data Availability

All data generated or analyzed during this study are included in this published article (Supplementary Data).

## Abbreviations

TTsupra: Thermal time constant at supra-optimal temperature
TTsub: Thermal time constant at sub-optimal temperature
θH: Hydrotime constant
θHTT: Hydrothermal time constant
T: Temperatures
GR: Germination rate
ψ: Water potential
PEG: Polyethylene glycol
GI: Germination index
SVI-I: Seed vigor index I
GRI: Germination rate index
GE: Germination energy
SVI-II: Seed vigor index II
MGT: Mean germination time
GP: Germination percentage
GRI: Germination rate index
T50%: Time to 50 percent germination, To Optimal temperature, Tb Base temperature, Tc ceiling temperature
